# miR-29a activates Hes1 by targeting Nfia in esophageal carcinoma cell line TE-1

**DOI:** 10.3892/ol.2014.2678

**Published:** 2014-11-06

**Authors:** CHANG LIU, PING DUAN, BO LI, CHUNTIAN HUANG, YING JING, WENHAI YAN

**Affiliations:** 1 Department of Preventive Medicine, Luohe Medical College, Luohe, Henan 462002, P.R. China; 2 Department of Basic Medicine, Zhengzhou University, Zhengzhou, Henan 450000, P.R. China; 3 Department of Oncology, The First Affiliated Hospital of Zhengzhou University, Zhengzhou, Henan, P.R. China

**Keywords:** microRNA, esophageal squamous cell carcinoma, notch, nuclear factor 1 A, cell growth

## Abstract

MicroRNA (miR)-29a has been associated with carcinogenesis in humans; however, its functional significance in esophageal squamous cell carcinoma (ESCC) is yet to be determined. In the present study, the expression of miR-29a was markedly downregulated in ESCC tissue and the ESCC TE-1 cell line, compared with normal esophageal tissue and cells. Furthermore, the present study identified that the forced expression of miR-29a in TE-1 cells significantly reduced cell proliferation and migration. miR-29a overexpression did not affect the expression of Notch1, however, it did increase the gene expression levels of hairy and enhancer of split 1 (Hes1), which is the key effector of the Notch signaling pathway. Direct targeting by miR-29a resulted in the downregulation of nuclear factor 1 A (Nfia), which represses the transcriptional activity of the Hes1 promoter. Furthermore, knockdown of Nfia increased Hes1 expression and inhibited cell growth in TE-1 cells. These results indicate that a low level of miR-29a expression is involved in ESCC tumorigenesis, and exogenous expression of miR-29a may repress cancer cell growth by downregulating Nfia and activating the Notch signaling pathway.

## Introduction

MicroRNAs (miRNAs) are a conserved class of endogenous non-coding small RNAs (length, 20–22 nt) that regulate gene expression at the post-transcriptional level. This predominantly occurs by binding to the 3′-untranslated region (UTR) mRNA of target genes, resulting in mRNA degradation and, thus, inhibition of translation (1–3). Recent studies have indicated that miRNA expression may be important in in the progression and outcome of various diseases (4,5). Although studies investigating miRNA expression profiles in esophageal carcinoma have been conducted (6), there remains little information available regarding specific miRNA expression patterns and their roles in esophageal squamous cell carcinoma (ESCC).

The Notch signaling pathway is important in stem cell maintenance and angiogenesis, as well as decisions regarding cell fate in cancer (7). Notch signaling is important for esophageal epithelial homeostasis, for example Notch signaling regulates cell proliferation within the squamous epithelia (8,9). Following activation of the Notch receptor, the Notch intracellular domain (NICD) is cleaved, released and translocated to the nucleus, where, in association with recombination signal binding protein for immunoglobulin KJ, it induces the expression of downstream target genes, including the hairy and enhancer of split (HES)/hairy and enhancer of split related with YRPW motif family of transcription factors (10).

The present study investigated the expression of miR-29a in ESCC and the role of miR-29a in cell growth and migration of the ESCC TE-1 cell line. Furthermore, the mechanisms of miR-29a modulation during TE-1 cell growth were evaluated.

## Materials and methods

### Cell lines

Primary cultures of normal esophageal epithelial cells (NEECs) were established from fresh biopsies of non-cancerous esophageal tissues, in accordance with a previous study (11). The NEECs and ESCC cells were cultured in keratinocyte serum-free medium (Gibco Life Technologies, Carlsbad, CA, USA) supplemented with 40 μg/ml bovine pituitary extract (Gibco Life Technologies), 1.0 ng/ml epidermal growth factor (Invitrogen Life Technologies, Inc., Carlsbad, CA, USA) 100 U/ml penicillin (Gibco Life Technologies) and 100 μg/ml streptomycin (Gibco Life Technologies), at 37°C and an atmosphere of 5% CO_2_. The ESCC TE-1 cell line was obtained from the Cell Bank of Type Culture Collection of the Chinese Academy of Sciences (Shanghai, China), and grown in RPMI-1640 medium (Invitrogen Life Technologies, Inc.) supplemented with 10% fetal bovine serum (FBS; Invitrogen Life Technologies), 100 μg/μl streptomycin (Gibco Life Technologies) and 100 μg/μl penicillin (Gibco Life Technologies) in a humidified incubator containing 5% CO_2_ at 37°C.

### Patient information and tissue specimens

The present study included nine ESCC tissue samples, which were histopathologically and clinically diagnosed at The First Affiliated Hospital of Zhengzhou University (Zhengzhou, China) in 2011, as well as nine adjacent non-cancerous esophageal tissue samples. Written informed consent was obtained from each patient and the present study was approved by the ethics committee of Zhengzhou University.

### Reverse transcription-quantitative polymerase chain reaction (RT-qPCR)

Total RNA, including miRNA, was extracted using the mirVana™ miRNA Isolation kit (Ambion Life Technologies, Carlsbad, CA, USA), in accordance with the manufacturer’s instructions. miR-29a was detected using the RT2 miRNA First Strand kit (SA Biosciences, Frederick, MD, USA) and specific miR-29a and U6 primers (Qiagen, Shanghai, China) were used for RT-qPCR. The relative expression of miR-29a was calculated using the comparative 2−ΔΔCt method. cDNA was synthesized using M-MLV reverse transcriptase (Promega, Madison, WI, USA) following standard protocols. Briefly, 3 μg total RNA and 2 μM oligo-dT primer (Promega) were added to RNase-free H_2_O. The RNA primer mixture was incubated at 65°C for 5 min then placed on ice for 3 min. Next, 0.5 mM dNTP mix (Promega), 10 μl M-MLV 5X reaction buffer (Promega), 400 U M-MLV reverse transcriptase and RNase-free H_2_O were added to the RNA primer mixture. The conditions for reverse transcription were as follows: 30°C for 10 min, 42°C for 1 h and 95°C for 10 min. The EzOmics SYBR qPCR kit was purchased from Biomics USA Inc., (Palo Alto, CA, USA), which included 5 μl cDNA template and 15 μl reaction mixture, containing 10 μl 2X SYBR Green mix, 0.5 μM forward primer, 0.5 μM reverse primer and RNase-free H_2_O. A qRT-PCR detection system (Applied Biosystems Life Technologies, Foster City, CA, USA) was used to perform RT-PCR. The amplification procedure was as follows: 94°C for 5 min, followed by 30 cycles at 94°C for 30 sec, 58°C for 30 sec for Nfia and glyceraldehyde 3-phosphate dehydrogenase (GAPDH), 55°C for 30 sec for Notch1 and Hes1, and 72°C for 10 min. The primer sequences of the genes were synthesized by Sangon Biotech (Shanghai) Co., Ltd., (Shanghai, China) and the sequences were as follows: Forward, 5′-ACCAGCTCAAAAAACCTGTGGA-3′ and reverse, 5′-TGTTGTGAAACGAAACACCCC-3′ for Nfi; forward, 5′-CACTGTGGGCGGGTCC-3′ and reverse, 5′-GTTGTATTGGTTCGGCACCAT-3′ for Notch1; forward, 5′-GTGTTAACGCCCTCACACG-3′ and reverse, 5′-TGGGAGGCAGACTAGCAGAG-3′ for (Hes1) and; forward, 5′-TTCAGCTCTGGGATGACCTT-3′ and reverse, 5′-TGCCACTCAGAAGACTGTGG-3′ for GAPDH. The mRNA expression of each gene was normalized to that of GAPDH. The relative mRNA expression was calculated using the comparative Ct method (2^−ΔΔCt^).

The nuclear factor 1 A (Nfia) mRNAs were determined using SYBR^®^-Green real-time PCR assay. The PCR primers were as follows: Sense, 5′-ACCAGCTCAAAAAACCTGTGGA-3′ and anti-sense, 5′-TGTTGTGAAACGAAACACCCC-3′ for Nfia; sense, 5′-CACTGTGGGCGGGTCC-3′ and anti-sense, 5′-GTTGTATTGGTTCGGCACCAT-3′ for Notch1; sense, 5′-GTGTTAACGCCCTCACACG-3′ and anti-sense, 5′-TGGGAGGCAGACTAGCAGAG-3′ for Hes1; and sense, 5′-TTCAGCTCTGGGATGACCTT-3′ and anti-sense, 5′-TGCCACTCAGAAGACTGTGG-3′ for GAPDH. GAPDH was used to normalize the mRNA expression.

### Lentiviral-mediated miR-29a overexpression and Nfia knockdown in mesenchymal stem cells

The miR-29a precursor vector and scramble plasmid were obtained from GeneCopoeia Inc., (cat no. RmiR6139; Guangzhou, China) and contained the puromycin selection marker. The lentivirus containing miR-29a precursor was generated using the Lenti-Pac™ Human Immunodeficiency Virus Expression Packaging kit (GeneCopoeia Inc.). Briefly, the DNA-EndoFectin complex was formed by adding 2.5 μl lentiviral miR-29a precursor expression plasmid/scramble plasmid to 5.0 μl EndoFectin™ Lenti transfection reagent diluted into Opti-MEM^®^. Following a 20-min incubation at room temperature, the DNA-EndoFectin complex was added to a petri dish containing 293T cells that had been plated in Dulbecco’s modified Eagle’s medium (DMEM; Gibco Life Technologies) supplemented with 10% FBS and incubated in 5% CO_2_ at 37°C overnight. The culture medium was replaced with fresh DMEM supplemented with 5% FBS and TiterBoost™ reagent (1:500; GeneCopoeia, Rockville, MD, USA), and incubation was continued. The virus pseudovirus-containing culture medium was collected 48 h post-transfection, filtered and concentrated. For lentiviral transduction, TE-1 cells were incubated for 2 h at 4°C, and 1×10^6^ TE-1 cells were plated with 20 μl virus suspension and cultured in an atmosphere of 5% CO_2_ at 37°C for 48 h. Following incubation, 10 μg/ml puromycin (EMD Millipore, Billerica, MA, USA) was added to the cells, all of which were subsequently preserved in a medium containing puromycin (final concentration, 10 μg/ml). The TE-1 cells stably expressing the exogenous genes, miR-29a precursor or scramble, were termed miR-29a-TE-1 cells or scramble-TE-1 cells, respectively. The siRNA vector against Nfia and the scramble plasmid were obtained from GeneCopoeia, Inc.

### MTT assay

Cells were seeded onto 96-well plates at a density of 5×10^4^ cells/well in 100 μl medium. All cells were maintained in a humidified incubator at 37°C and an atmosphere of 5% CO_2_. MTT (20 μl; 5g/l) was added to each well of the microplate and a microplate reader (Anthros 2010; Biochrom Ltd., Cambridge, UK) was used to measure the absorbance at 570 nm. Following a 4-h incubation, the number of viable cells was measured. Five wells were counted at each time point and the mean was calculated.

### Flow cytometry

The percentage of sub-G1 population (apoptotic) cells and the cell cycle distribution were determined using flow cytometry, based on the relative DNA content as previously described (12). The data were analyzed using ModFit LT software (version 3.1; Verity Software House, Topsham, ME, USA).

### Colony formation assays

Cells were plated on 60-mm plates (0.5×10^3^ cells/plate) and cultured for 10 days. The colonies were stained with 1% crystal violet for 30 sec following fixation with 10% formaldehyde for 5 min.

### In vitro scratch assay

TE-1 cells (5×10^6^ cells/well) were seeded in a six-well plate and cultured overnight to reach a confluence of 90%. The following day, a scratch was made through the center of each well using a 200-μl pipette tip, creating an obvious open scratch or wound on the cells. The dislodged cells were removed by washing three times with the complete culture media, and the remaining cells were incubated under standard conditions. Migration into the open area was identified 72 h post-scratching. In addition, TE-1 cells were transfected with lentivirus containing a miR-29a precursor or control for four days then treated with 20 ng/ml epidermal growth factor (EGF; Sigma-Aldrich, St. Louis, MO, USA) for 16 hrs and seeded in six-well plates. After the cells had reached 90% confluence, a scratch was made in the monolayer.

### Western blot analysis

TE-1 cells were washed with phosphate-buffered saline (PBS; Gibco Life Technologies), then 200 μl/well cell lysis buffer (Beyotime Institute of Biotechnology, Haimen, China) and 1 mM phenylmethanesulfonyl fluoride were added. Next, the cell lysate was centrifuged twice at 2,000 × g for 20 min at 4°C and the supernatant was transferred to a clean tube. Protein concentration was measured using a BCA assay kit (Beyotime Institute of Biotechnology). Proteins were separated by SDS-PAGE and transferred to polyvinylidene difluoride membranes (Amersham Pharmacia Biotech Inc., Piscataway, NJ, USA). The membranes were blocked with 5% non-fat milk in PBS and Tween 20 (PBST; Beyotime Institute of Biotechnology) for 1 h at room temperature and incubated with polyclonal rabbit anti-goat Nfia (sc-30918; 1:1,000; Santa Cruz Biotechnology, Inc., Santa Cruz, CA, USA), polyclonal rabbit anti-goat Notch1 (sc-6014; 1:1,000; Santa Cruz Biotechnology, Inc.), polyclonal rabbit anti-goat Hes1 (sc-13842; 1:1,000; Santa Cruz Biotechnology, Inc.) or polyclonal rabbit anti-goat β-actin (sc-1616; 1:1,000; Santa Cruz Biotechnology, Inc.), at 4°C overnight. The membranes were washed with PBST three times for 5 min, then incubated with polyclonal horseradish peroxidase-conjugated rabbit anti-goat antibody (sc-2768; Santa Cruz Biotechnology, Inc.) for 1 h at room temperature. Enhanced chemiluminescence was performed according to the manufacturer’s instructions (Amersham Pharmacia Biotech Inc., Piscataway, NJ, USA).

### Vector construction and luciferase assay

pGL3-Nfia was generated by amplifying a 197-bp 3′UTR fragment of the Nfia gene containing the miR-29a binding site predicted using the TargetScan version 6.0 (http://www.targetscan.org/) and subsequently cloning it into the pmirGLO Dual-Luciferase miRNA Target Expression vector (Promega) at the *Nhe*I and *Sal*I (Takara Bio, Inc., Otsu, Japan) cleavage sites, immediately downstream of firefly luciferase. The primer sequences used for amplification were as follows: Sense, 5′-GCGCTAGCCAGCAAGCATTATGGTCAAACA-3′ and anti-sense, 5′-GCGTCGACGGAAGTCAGTGAGCAAGGGTAG-3′. (the restriction enzyme sites are underlined). The TE-1 cells were initially transfected with the miR-29a or scrambled virus for 4 days, and subsequently transfected with pmirGLO-Nfia using Lipofectamine 2000 (Invitrogen Life Technologies, Inc.). Luciferase activity was measured 24 h after transfection with pmirGLO-Nfia using the Dual-Glo™ Luciferase assay system (Promega). The Renilla luciferase activity served as the internal control.

### Statistical analysis

Statistical evaluation of the data was conducted using SPSS analysis software (version 13; SPSS Inc., Chicago, IL, USA) and comparisons were performed using the Wilcoxon signed-rank test or independent samples t-tests. P<0.05 was considered to indicate a statistically significant difference.

## Results

### miR-29a expression is downregulated in ESCC tissues and the ESCC TE-1 cell line

**Figure 1:**
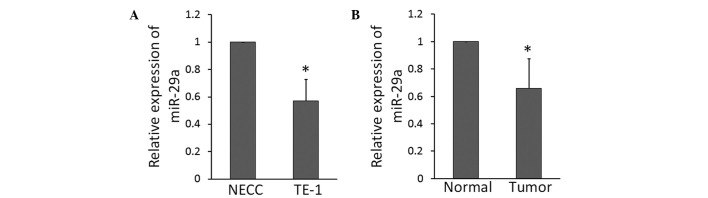
Expression levels of miR-29a are reduced in ESCC. (A) Expression of miR-29a in NEECs and ESCC TE-1 cells. Expression levels were normalized using U6 and error bars represent standard deviations calculated from three parallel experiments. (B) Expression of miR-29a in paired esophageal adjacent non-cancerous tissue (normal) and primary ESCC tissue (tumor) from the same patient. Expression levels were normalized with U6 and error bars represent standard deviations calculated from nine patients. ^*^P<0.05. miR, microRNA; ESCC, esophageal squamous cell carcinoma; NEEC, normal human esophageal epithelial cells.

RT-qPCR analysis revealed that the expression of miR-29a was significantly lower in the ESCC TE-1 cell line compared with in NEECs (Fig. 1A). To understand whether this miR-29a downregulation was clinically correlated with ESCC progression, a comparative analysis of miR-29a expression was conducted on paired primary cancerous tissue and adjacent non-cancerous tissue from nine cases of ESCC. RT-qPCR analysis revealed that the expression of miR-29a was significantly lower in the tumor tissue compared with the adjacent non-cancerous tissue (Fig. 1B).

### Overexpression of miR-29a reduces cell proliferation and inhibits the migration of ESCC cells

**Figure 2:**
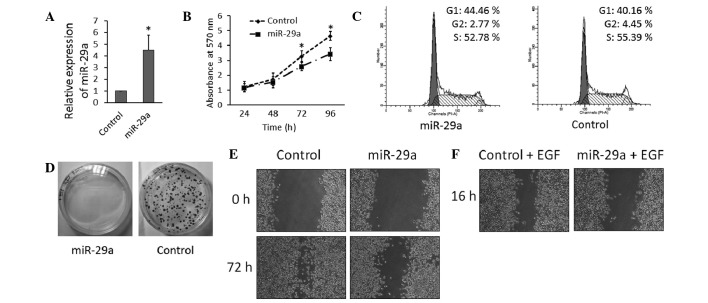
Overexpression of miR-29a in ESCC cells reduces cell proliferation and inhibits cell migration. (A) Expression levels of miR-29a were significantly increased in TE-1 cells following virus transfection, as analyzed by reverse transcription-quantitative polymerase chain reaction. ^*^P<0.05. (B) Overexpression of miR-29a inhibits TE-1 cell proliferation, as determined by MTT assay. (C) Overexpression of miR-29a inhibits TE-1 cell proliferation, as determined by three independent flow cytometry experiments (data are presented as the mean ± standard deviation). (D) Upregulation of miR-29a inhibits cell growth, as determined by colony formation assays. (E) Effect of miR-29a on cell migration in a long-interval scratch assay. TE-1 cells were transfected with lentivirus containing miR-29a precursor or control for four days, seeded in six-well plates and grown to confluence. A scratch was made in the cell monolayer and images were captured at 72 h (magnification, ×40). (F) Effect of miR-29a on cell migration in a short-interval scratch assay. TE-1 cells were transfected with lentivirus containing miR-29a precursor or control for four days, treated with EGF (20 ng/ml), seeded in six-well plates and grown to confluence. A scratch was made in the cell monolayer and images were captured 16 h after EGF stimulation (magnification, ×40). miR, microRNA; ESCC, esophageal squamous cell carcinoma; EGF, epidermal growth factor.

To investigate the biological role of miR-29a in ESCC progression, the ESCC TE-1 cell line was transfected with lentivirus containing the miR-29a precursor or scramble control. The overexpression of miR-29a reduced cell proliferation and resulted in an accumulation of G0/G1 phase cells, indicating that miR-29a induces G0/G1 arrest in TE-1 cells (Fig. 2A, B, C). Furthermore, the ability of miR-29a to regulate cell proliferation was indicated by a colony formation assay, which demonstrated that miR-29a significantly decreased the colony formation ability of TE-1 cells (Fig. 2D). Long intervals are required to measure cell migration or to observe healing of scratches in cancer cell monolayers (13); after 72 h, overexpression of miR-29a significantly inhibited the ability of TE-1 cells to heal following scratching (Fig. 2E). To investigate the effect of miR-29a on cell migration over a shorter interval (16 h), thereby minimizing the confounding effect of cell proliferation, TE-1 cell migration was stimulated with EGF (Sigma-Aldrich). The motility of TE-1 cells overexpressing miR-29a was significantly slower compared with that of the control (Fig. 2F).

### Overexpression of miR-29a upregulates Hes1 and downregulates Nfia

**Figure 3:**
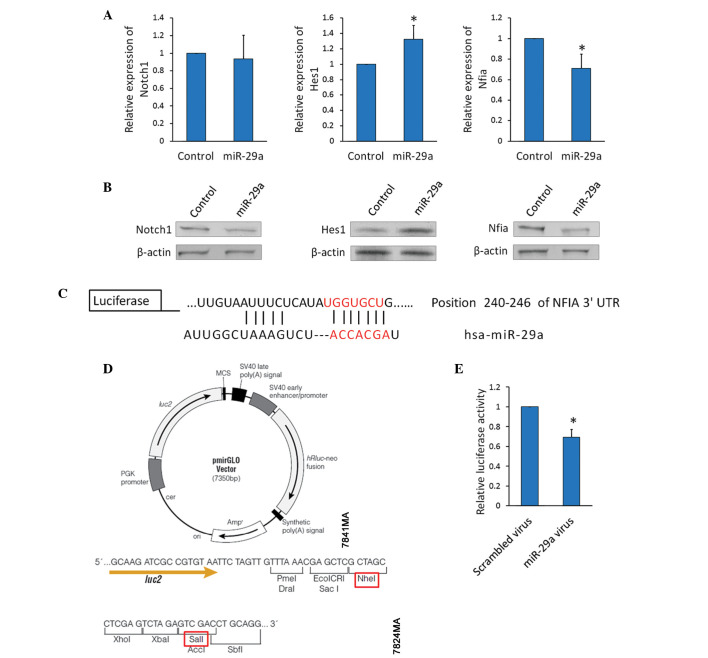
Overexpression of miR-29a upregulates Hes1 and downregulates Nfia. (A) Notch1, Hes1, and Nfia mRNA expression levels were detected using reverse transcription-quantitative polymerase chain reaction in TE-1 cells transfected with the miR-29a precursor or control virus.^*^P<0.05. (B) Notch1, Hes1, and Nfia protein expression levels were detected using western blot analysis in TE-1 cells transfected with the miR-29a precursor virus or control virus. (C) TargetScan prediction of the miR-29a binding site within Nfia mRNA. (D) Map of the pmirGLO luciferase reporter vector. The red rectangles indicate the restrictive endonucleases used for cloning. (E) Luciferase activity assay: TE-1 cells were transfected with the miR-29a precursor or scrambled virus for four days, transfected with the reporter vectors for 24 h, and harvested. Protein extracts were prepared and assayed for firefly and Renilla luciferase activity, and firefly luciferase activity was normalized to Renilla luciferase activity. Data are presented as the mean ± standard deviation from three independent experiments. ^*^P<0.05. miR, microRNA; Hes1, hairy and enhancer of split 1; Nfia, nuclear factor 1 A; UTR, untranslated region.

RT-qPCR and western blot analysis were used to determine the expression of Notch1 and Hes1 in TE-1 cells. The mRNA (Fig. 3A) and protein (Fig. 3B) expression levels of Notch1 did not significantly change in miR-29a-overexpressing-TE-1 cells; however, Hes1, located downstream of the Notch signaling pathway, was significantly upregulated and Nfia, which reduces Hes1 expression by repressing Hes1 promoter transcriptional activity, was downregulated by miR-29a overexpression.

Investigations into the mechanism for the downregulation of Nfia expression by miR-29a resulted in the identification of a potential binding site for miR-29a at position 240–246 of the Nfia 3′UTR mRNA (Fig. 3C). It was hypothesized that miR-29a represses Nfia expression via this site; thus, reporter vectors, containing luciferase complementary DNA followed by the Nfia 3′UTR, were constructed (Fig. 3D). TE-1 cells were transfected with the miR-29a precursor or scrambled virus for 4 days, and the resultant miR-29a-overexpressing TE-1 cells were then transfected with the reporter vectors. Luciferase activity was significantly decreased in the miR-29a-overexpressing reporter vector (Fig. 3E). This demonstrates that miR-29a directly inhibited Nfia expression by binding to its mRNA.

### Knockdown of Nfia increases Hes1 expression and inhibits the growth of TE-1 cells

**Figure 4:**
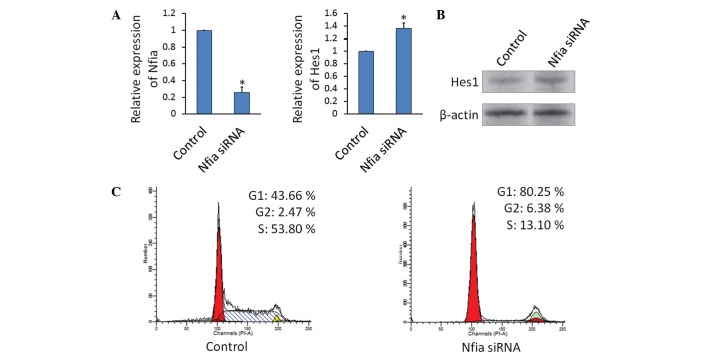
Knockdown of Nfia in TE-1 cells increases Hes1 expression levels and inhibits cell growth. (A) Reverse transcription-quantitative polymerase chain reaction was performed to determine Nfia and Hes1 mRNA expression levels in Nfia-knockdown TE-1 cells and control cells. ^*^P<0.05. (B) Western blot analysis was performed to determine Hes1 protein expression levels in Nfia-knockdown TE-1 cells and control cells. (C) Knockdown of Nfia inhibits TE-1 cell proliferation, as determined by flow cytometry. Data are presented as the mean ± standard deviation from three independent experiments. Nfia, nuclear factor 1 A; Hes1, hairy and enhancer of split 1.

TE-1 cells were transfected with lentivirus-containing siRNA against Nfia or the scramble RNA control. The knockdown of Nfia significantly increased the gene (Fig 4A) and protein (Fig 4B) expression levels of Hes1 in TE-1 cells. Furthermore, the knockdown of Nfia reduced cell proliferation and resulted in an accumulation of cells in the G0/G1 phase (Fig. 4C).

## Discussion

miRNAs are small, endogenous, non-coding RNA molecules that regulate the expression of protein-coding genes. miRNAs appear to affect numerous biological processes and diseases (14,15). Although the mechanisms of various miRNAs remain poorly understood*,* and the existence of specific miRNAs remains controversial, recent studies have provided significant insights the miR-29 family, including its biology and relevance to cancer (16,17). Mature miR-29s in humans include hsa-miR-29a, -29b, and -29c, which are highly conserved in humans, mice and rats (17). All mature miR-29s share identical sequences at nucleotide positions 2–7, the seed region that is key in determining which protein-coding genes an miRNA targets (17).

The downregulation of miR-29 family members has been correlated with various types of cancer, including leukemia (18,19), melanoma (20), and liver (21), colon (22), cervical (23) and lung (24,25) cancer; thus, miR-29s may serve as tumor suppressors. In the present study, miR-29a was initially demonstrated to be downregulated in ESCC tissue and ESCC TE-1 cells. It has been reported that the dysfunction of miR-29a results in abnormal cell growth (16,26). In the current study, in order to investigate the role of miR-29a in ESCC, an assay of the cell cycle of TE-1 cells was conducted following pre-miR-29a transfection-induced miR-29a overexpression. Overexpression of miR-29a markedly arrested the cell cycle in the G0/G1 transition, indicating that miR-29a predominantly regulates ESCC cell proliferation through the modulation of cell cycle progression. In numerous studies, the downregulation of miR-29 has been shown to correlate with the motility and migration of carcinoma cells (27–30). In the present study, the overexpression of miR-29a reduced cell migration in TE-1 cells. These results indicate that miR-29a downregulation results in uncontrolled cell cycle progression in ESCC cells and is involved in ESCC tumorigenesis.

The Notch signaling pathway is a highly conserved cell signaling system, present in the majority of multicellular organisms. The Notch signaling pathway is involved in cell fate decisions during normal development and during the development of various types of cancer (7). The Notch signaling pathway is present in all metazoans and includes four different Notch receptors, termed NOTCH1, NOTCH2, NOTCH3 and NOTCH4. When the Notch signaling pathway is activated, the intracellular domain is released and enters the cell nucleus to modify gene expression, including that of Hes-1. In the present study, it was identified that miR-29a overexpression did not affect Notch1 gene expression levels but did increase the expression levels of its downstream gene, Hes1. Unlike the majority of signaling pathways, Notch signaling can be oncogenic or tumor-suppressive, depending on the cellular context (7). In ESCC cells, Ohashi *et al* (31) reported that downregulation of the Notch signaling pathway resulted in the attenuation of squamous cell differentiation and the enhancement of an invasive subset of ESCCs, indicating that Notch may act as tumor suppressor in ESCCs. Thus, the present study proposes that the overexpression of miR-29a reduces cell growth and migration by activating the Notch signaling pathway in TE-1 cells.

Furthermore, the present study investigated the mechanisms by which miR-29a modulates Hes1 gene expression levels and identified that the transcription factor Nfia may be key in this progression. Nfia belongs to the nuclear factor I (NFI) family of site-specific DNA-binding proteins, which are important in various fields, including animal physiology, biochemistry and pathology. NFI proteins have been associated with changes in the growth state of cells and with a number of oncogenic processes and disease states. Previous studies demonstrated that Nfia reduces the expression of Hes1 by repressing transcriptional activity under the control of the Hes1 promoter (32). In the present study, miR-29a overexpression decreased Nfia gene and protein expression levels in TE-1 cells. In addition to the gene prediction analysis conducted by TargetScan, this observation clarified that Nfia is the direct target gene of miR-29a. In order to verify the role of Nfia in TE-1 cells, Nfia was knocked down; this resulted in increased Hes1 gene and protein expression levels and inhibited the TE-1 cell growth. Thus, the results of the present study demonstrated that the overexpression of miR-29a downregulated Nfia, which in turn increased the Hes1 expression in TE-1 cells. Therefore, the present study proposes that Notch pathway-targeted therapy using miR-29a may be a promising treatment for ESCC.

In conclusion, the present study proposes that miR-29a is an important miRNA that negatively regulates the Notch signaling pathway by targeting Nfia and modulating Hes1 expression. The study demonstrated that miR-29a was poorly expressed in ESCC and involved in ESCC tumorigenesis. Furthermore, data from the present study indicates that overexpression of miR-29a inhibits the growth of TE-1 cells, supporting the therapeutic potential of this novel miRNA in ESCC.
